# Role of the Benzothiadiazole Unit in Organic Polymers on Photocatalytic Hydrogen Production

**DOI:** 10.1021/jacsau.3c00681

**Published:** 2024-01-13

**Authors:** Martin Axelsson, Ziyang Xia, Sicong Wang, Ming Cheng, Haining Tian

**Affiliations:** † Department of Chemistry-Ångström Laboratory, Uppsala University, Uppsala 75120, Sweden; ‡ Institute for Energy Research, Jiangsu University, Zhenjiang 212013, China

**Keywords:** photocatalysis, polymer dots. benzothiadiazole, hydrogen, protonation

## Abstract

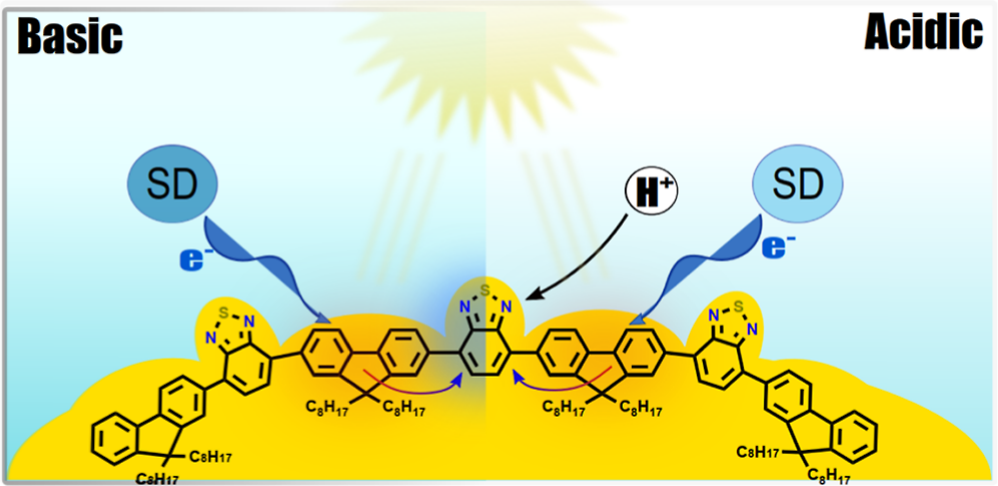

Organic polymers based on the donor–acceptor structure are a promising class of efficient photocatalysts for solar fuel production. Among these polymers, poly(9,9-dioctylfluorene-alt-1,2,3-benzothiadiazole) (PFBT) consisting of fluorene donor and benzothiadiazole acceptor units has shown good photocatalytic activity when it is prepared into polymer dots (Pdots) in water. In this work, we investigate the effect of the chemical environment on the activity of photocatalysis from PFBT Pdots for hydrogen production. This is carried out by comparing the samples with various concentrations of palladium under different pH conditions and with different sacrificial electron donors (SDs). Moreover, a model compound 1,2,3-benzothiadiazole di–9,9-dioctylfluorene (BTDF) is synthesized to investigate the mechanism for protonation of benzothiadiazole and its kinetics in the presence of an organic acid–salicylic acid by cyclic voltammetry. We experimentally show that benzothiadiazole in BTDF can rapidly react with protons with a fitted value of 0.1–5 × 10^10^ M^–1^ s^–1^ which should play a crucial role in the photocatalytic reaction with a polymer photocatalyst containing benzothiadiazole such as PFBT Pdots for hydrogen production in acidic conditions. This work gives insights into why organic polymers with benzothiadiazole work efficiently for photocatalytic hydrogen production.

## Introduction

Filling the demands of being low-cost and tunable, with excellent light-absorbing properties, organic polymers have risen as a type of promising photocatalysts for solar fuel production.^1,2^ A common configuration of polymeric photocatalysts is based on so-called donor–acceptor structures with alternating electron-rich and electron-poor units to facilitate exciton formation from locally excited states.^3−5^ Since this type of polymer has been widely used in photodiodes as well as organic solar cells, their photophysical and semiconductive properties are widely studied.^6−8^ As photocatalysts, similarly, the polymers have been mostly tuned to maximize their physical and optical properties, while studies of how and where the catalytic chemistry occurs are still few.

Many polymers and polymer nanoparticles such as Pdots have shown that they have satisfactory photocatalytic hydrogen production even without the addition of a cocatalyst.^9−15^ Since most of the polymers are synthesized by a coupling reaction in the presence of a Palladium(Pd)-catalyst,^16,17^ some Pd is typically trapped in the polymers and acts as cocatalysts. Kosco et al. have studied the effect of residual Pd remaining from synthesis on the performance of photocatalytic hydrogen evolution in (poly(9,9-dioctylfluorene-*alt*-2,1,3-benzothiadiazole)) (F8BT or PFBT) and found that the photocatalytic activity has been completely inhibited when the polymer has less than 1 ppm Pd in the systems with 30% V of diethyl amine (DEA) as the sacrificial donor (SD).^18^ They also found that when the Pd content reached 40 ppm, the polymer could still produce a significant amount of hydrogen.^18^ However, the effect of the Pd cocatalyst seems to vary substantially from polymer to polymer. Some polymers with a decent amount of Pd show very low or absent activity for photocatalytic hydrogen production.^13,14^ It implies that the polymer structure, especially the acceptor unit used in the polymer, should play an important role in the photocatalytic reaction even if the residual Pd is the catalytic site since the acceptor unit is where the electron is concentrated in an excited or reduced polymer.

**Figure 1:**
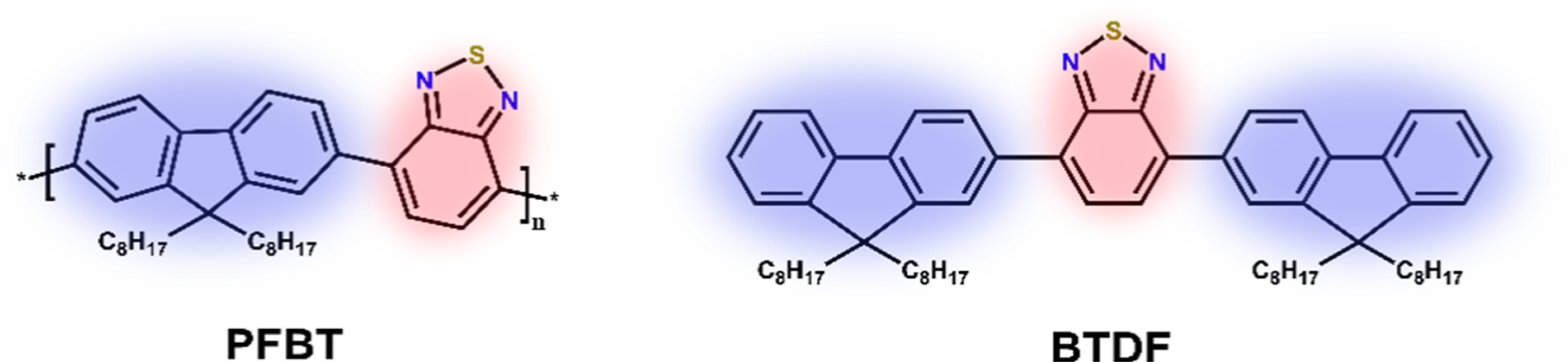
Chemical structures of PFBT and BTDF.

Recently, Hillman et al. investigated the importance of sulfone units normally used in some polymers on photocatalytic hydrogen production and found that sulfone units play important roles in light absorption;^19^ hole transfer to triethylamine (TEA) SD, and electron transfer to residual palladium.^1^ Benzothiadiazole (BT) unit is also a common acceptor used in polymers that have shown good photocatalytic performance.^13,20−22^ Recently, we have shown that a BT analogue benzothiadiazole dicarbonitrile (BTDN) could generate hydrogen on a glassy carbon electrode during the electrocatalytic conditions and the protonated intermediate species were studied.^23^ The highest activity of photocatalytic hydrogen generation with polymers or Pdot photocatalysts involving BT units has been reported in acidic conditions with ascorbic acid as the SD. Therefore, protonation of the BT unit probably is an important step for photocatalytic reaction. This motivated us to study the role of BT in addition to that of an electron acceptor during the photocatalytic reaction. PFBT polymer and a model compound (2,1,3-benzothiadiazole di-9,9-dioctylfluorene) (BTDF) as shown in Figure 1 are therefore selected in this work.

## Experimental Section

### Materials

PFBT was purchased from Ossila. PFBT from the same batch was treated with Brilliant Matters to remove palladium until it was below the detection limit of 10 ppm. Polystyrene grafted with carboxy-terminated poly(ethylene oxide) (PS–PEG-COOH, backbone chain Mw 8500 g mol^–1^, graft chain Mw 4.6 kDa, total chain 36,500 g mol^–1^) was purchased from Polymer Source Inc. All solvents and other chemicals are purchased from Sigma-Aldrich and used as received unless stated otherwise.

### Pdot Preparation

PFBT Pdots were prepared using the nanoprecipitation method, where a 5:1 mixture of PFBT (40 μg mL^–1^) and PS–PEG-COOH (8 μg mL^–1^) was dissolved in THF. The THF phase was then quickly stirred into H_2_O with a 1:2 ratio (V/V) while in an ultrasonication bath and then left in the bath for 10 min. Afterward, the THF was left to evaporate in ambient conditions for 2 days. Pdots with different samples are compared by UV–vis absorption spectroscopy (Figure S3) and dynamic light scattering to obtain similar absorption intensity and particle size, respectively.

### Photocatalytic Hydrogen Evolution

The photocatalytic hydrogen evolution experiments were performed in 9 mL gastight vials with 3 mL of 20 μg mL^–1^ Pdot suspension. A 1.2 M ascorbic acid solution was prepared separately and modified to pH 4 with 3 M NaOH. The Pdot suspension and ascorbic acid solution were degassed separately with argon gas, finally, 0.6 mL of the ascorbic acid was added to the Pdot suspension vial to a total of 3.6 mL. The vials were then illuminated with an LED PAR38 lamp (17 W, 5000 K, Zenaro Lighting GmbH, λ > 420 nm) used as the light source, in a black box removing any stray light. The light intensity on the illuminated area of the vial is 50 mW cm^–2^, which is measured and then calibrated with a power sensor (Thorlabs S120C, Si, 400–1100 nm, 50 mW connected to a PM100D console). The hydrogen was measured with a Gas chromatograph (PerkinElmer LLC, MA) calibrated from pure hydrogen injections. The H_2_ was sampled by removing 100 μL of gas from the headspace of the vial with a gastight Hamilton needle. The air was kept out of the vials by covering needle pinholes in the septum with Play-Doh clay (Hasbro, Inc.) as the needles were retracted.

### BTDF Synthesis

The synthetic route is shown in Scheme S1. 4,7-Dibromo-2,1,3-benzothiadiazole (compound 1) (1.00 g, 3.40 mmol), 2-(9,9-dioctyl-9*H*-fluoren-2-yl)-4,4,5,5-tetramethyl-1,3,2-dioxaborolane (compound 2) (4.22 g, 8.16 mmol), Pd(PPh_3_)_4_ (0.12 g, 0.10 mmol), Aliquat 336 (0.069 g, 0.17 mmol), K_2_CO_3_ (3.76 g, 27.22 mmol), H_2_O (30 mL), and toluene (90 mL) were placed in a round-bottom flask. The reaction mixture was stirred at 110 °C under nitrogen for 24 h. After the reaction, the solution was cooled to room temperature, and it was then extracted with dichloromethane, the two phases were separated, and the water phase was extracted twice with dichloromethane. The combined organic extracts were washed three times with water, dried over Na_2_SO_4_, evaporated, and purified with column chromatography (eluding with petroleum ether/dichloromethane, 5/1 v/v) to give 2.88 g of BTDF at a 92% yield. 1H NMR (CDCl_3_, 400 MHz) δ 8.03 (dd, *J* = 7.9, 1.6 Hz, 2H), 7.96 (d, *J* = 1.6 Hz, 2H), 7.87 (d, *J* = 8.3 Hz, 4H), 7.80–7.73 (m, 2H), 7.41–7.31 (m, 6H), 2.04 (tq, *J* = 13.3, 5.4, 4.2 Hz, 8H), 1.21–1.05 (m, 40H), 0.80 (t, *J* = 6.9 Hz, 20H). 13C NMR (CDCl_3_, 101 MHz): δ 14.09, 22.64, 23.94, 29.27, 29.75, 30.12, 31.85, 40.34 55.27, 119.74, 119.99, 122.99, 123.96, 126.88, 127.29, 127.90, 128.18, 133.64, 136.22, 140.71, 141.38, 151.13, 151.36, 154.42. HRMS-EIS (m/z): [M + H]^+^ calcd for (C_64_H_84_N_2_S), 913.4500; found, 913.6428. 13C NMR (CDCl_3_): δ 154.4, 151.4, 15.1.1, 141.4, 140.7, 136.2, 133.6, 128.2, 127.9, 127.3, 126.9, 124.0, 123.0, 120.2, 119.7, 55.3, 40.3, 31.8, 30.1, 29.8, 29.3, 23.9, 22.6, 14.1.

### Cyclic Voltammetry

All cyclic voltammetry in organic solvents was performed in a ⌀ 2.5 × 5 cm cylindrical glass cell. The solvent used was THF [>99.7% unstabilized (HPLC grade)] kept dry over 3 Å molecular sieves, with 0.2 M recrystallized tetrabutylammonium hexafluorophosphate (TBA PF6) as the supporting electrolyte. As working electrodes, either a ⌀ 3 mm glassy carbon disk or a ⌀ 3 mm palladium disk electrodes were used and both were polished with a 0.05 μM Al particle paste in-between measurements, as a counter electrode a Pt wire was used. As a reference electrode, a silver-wire (Ag/Ag^+^) pseudo reference was used in THF with the electrolyte in a glass tube connected to the solution with a porous Vycore frit; the potential is then confirmed with ferrocene as an internal standard. The water measurements were performed in a ⌀ 1.5 cm × 3 cm cylindrical cell with 50 mM KCl and 50 mM KH_2_PO_4_/K_2_HPO_4_ as electrolytes. The same working and counter electrodes were used as in the THF experiments but as a reference, an Ag/AgCl reference electrode was used. Highly concentrated samples (220 μg mL^–1^) of Pdots were used under the aqueous conditions to enhance the signal. The 20 μg mL^–1^ Pdots sample was concentrated from 10 mL by centrifugal filtration with an Amicron Ultra-15 10k centrifugal filter at 5000 rpm for 12 min, leaving 0.9 mL of Pdot solution.

### Simulations

Electrochemical simulations were performed in DigiElch 8FD with the basic parameters *k*_s_ = 0.0012 cm s^–1^ (rate constant for heterogeneous electron transfer), α = 0.5 (the transfer coefficient), and DBTDF = 3.2 × 10^–7^, DSAL = 1.5 × 10^–7^ (the diffusion coefficients with all protonated intermediates set to DBTDF) extracted from CVs of pristine BTDF in THF.

## Results and Discussion

### Condition Dependency on H_2_ Evolution from Pdots

The photocatalytic proton reduction hydrogen evaluation of Pdots is considered a half-reaction of water splitting, and the catalytic systems are normally optimized depending on the SD used in the system. However, the SD used for the study also dictates what pH the system can operate in since the highest activity of the SD is dependent on the pH of the solution.^24^ The pH of the catalytic system certainly has a significant effect on the catalytic performance since both the driving force to reduce protons^25^ and the catalytic mechanism can be affected by the presence of protons and the protic activity.^26^ DEA and ascorbate are the two SDs that have been commonly used in the majority of the literature for photocatalysis with PFBT Pdots and are therefore chosen in this study. DEA was reported at a basic condition, while ascorbate was reported at an acidic condition.^12,13,18,27^ Therefore, we first compared the photocatalytic hydrogen production of PFBT Pdots with different residual Pd, under these two conditions.

The hypothesis is that if protonation of the BT unit is involved in the photocatalytic reaction, it would be more likely to occur under acidic conditions. At the same time, we also checked the effect of the Pd amount in PFBT polymers on photocatalysis at different pH values since the number of active sites should have a large impact on catalytic activity. To identify the difference in photocatalytic performance, two PFBT samples with different Pd amounts, 1000 ppm, and less than 10 ppm of Pd (below the detection limit), were chosen in this study. The Pdots from two PFBT samples were prepared as similarly as possible and then the photocatalytic experiments were carried out at two conditions: 0.2 M ascorbic acid with pH = 4^12,13,28,29^ and 0.2 M DEA with pH = 13.5 (ca. 11 vol % as compared to the 30 vol % used in the reference).^18,27^ Under these two reaction conditions, there is almost a 10 orders of magnitude difference in proton concentration in the ascorbate condition (0.1 mM protons) as compared to that of the DEA condition (50 fM protons). pH difference between 13.5 (DEA) and 4 (ascorbic acid) also leads to a large difference in driving force of 0.56 V for proton reduction.

**Figure 2:**
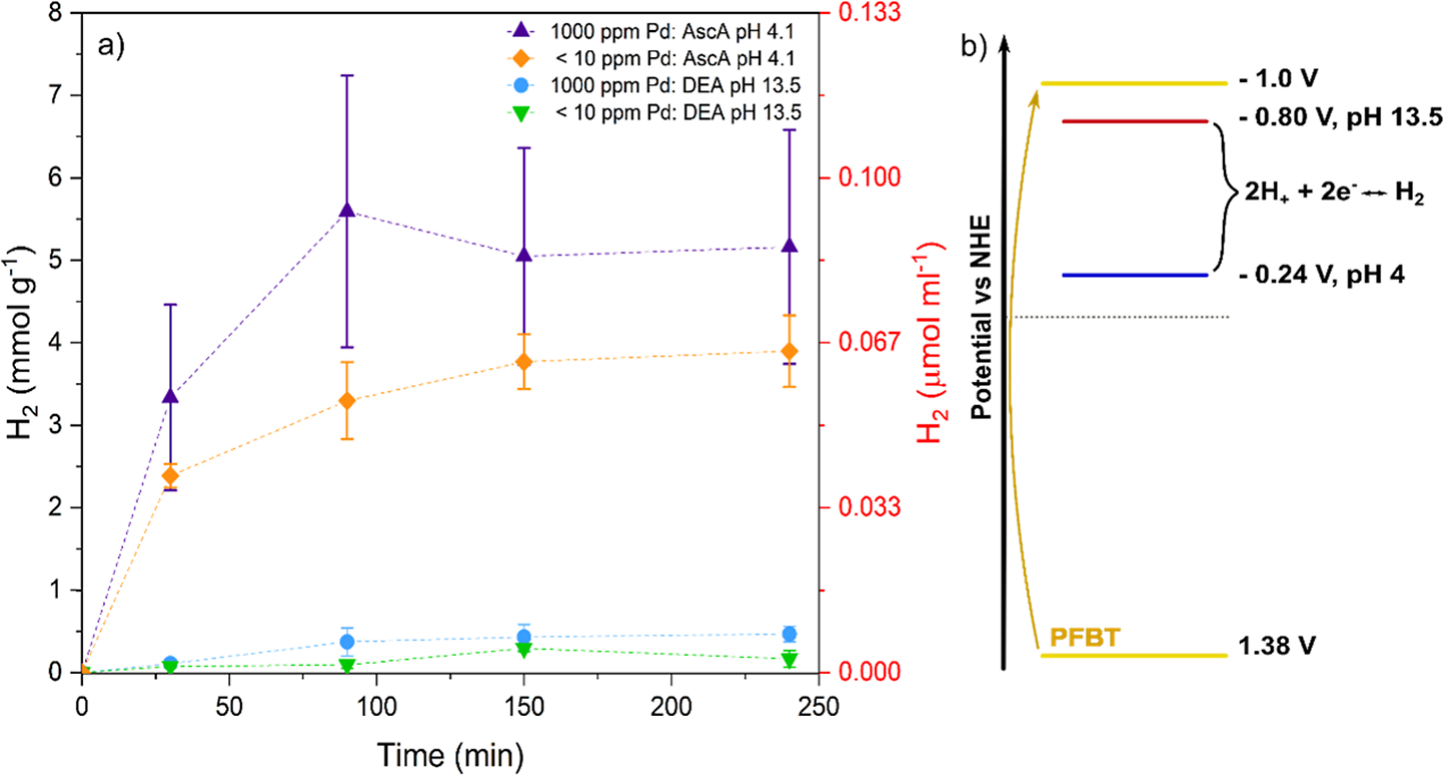
Comparison of different pH conditions and Pd contents for photocatalytic hydrogen production. (a) Photocatalytic hydrogen production under various conditions of hydrogen evolution from PFBT Pdots, ascorbate and high Pd content (purple triangles), ascorbate low Pd content (orange squares), DEA and high Pd content (blue circles), and DEA and low Pd content (green triangles). (b) Scheme of the energy levels of PFBT and thermodynamic potentials for proton reduction at two different pH values.

As shown in Figure 2b, at pH 13.5 in DEA, the driving force for PFBT polymer to reduce the proton is only 0.2 V. The photocatalytic hydrogen evolution experiment (Figure 2a) shows a clear trend where the samples with acidic conditions significantly outperform the basic ones. None of the samples under basic conditions showed satisfactory hydrogen production (Figure S4). In acidic conditions, the sample with 1000 ppm of Pd showed a slightly higher photocatalytic performance with an initial hydrogen generation rate of 7 mmol g^–1^ h^–1^ than that of the one with less than 10 ppm of Pd, with an initial hydrogen generation rate of 5 mmol g^–1^ h^–1^. The large difference in driving force between the two pH conditions seems to be a major factor. Experiments with a systematical pH dependence for hydrogen production are useful to determine the change of photocatalytic activity of PFBT Pdots under various pH conditions. However, since SDs reductive activity is dependent on pH it is impossible to vary the pH without affecting the availability of sacrificial electrons in the photocatalytic experiment. Instead, we, therefore, carried out electrocatalytic experiments under various pH conditions, to decouple the electron availability from the sacrificial donor and pH condition.

### Electrocatalysis and pH Dependence

**Figure 3:**
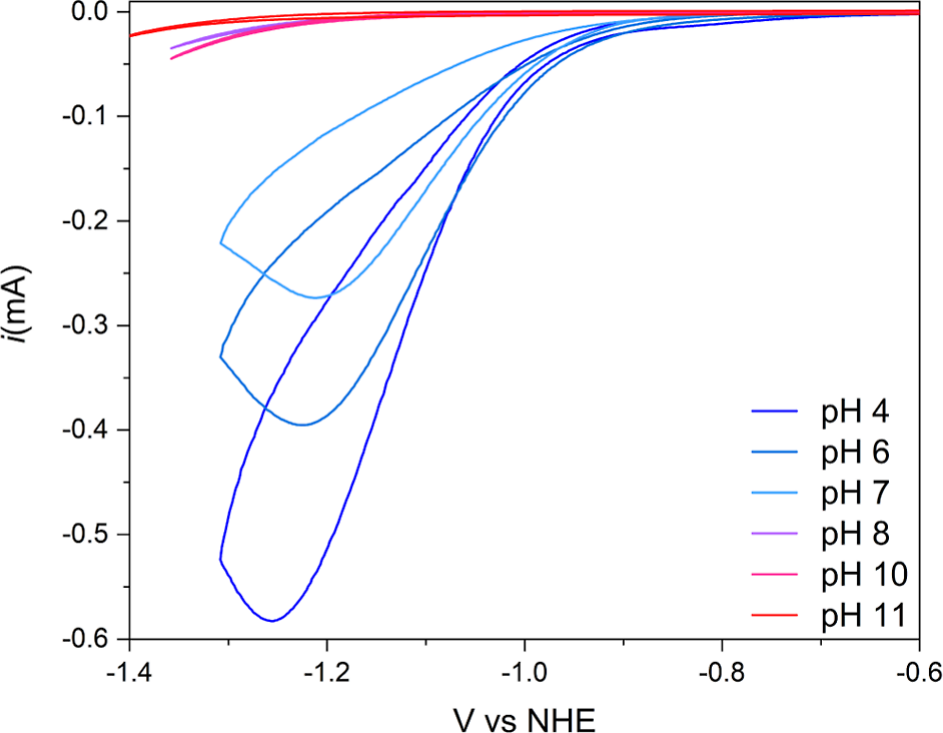
CVs demonstrate the pH dependence of electrocatalytic hydrogen evolution from PFBT Pdots, in 50 mM phosphate buffer and 50 mM KCl as the supporting electrolyte, showing the decreasing catalytic current at 50 mV s^–1^ from pH 4 to pH 7 with a collapse of current between pH 7 and pH = 8.

We have previously demonstrated that PFBT Pdots could be active to generate hydrogen evolution on a glassy carbon electrode under acidic conditions.^12^ Herein, we performed the electrocatalysis of Pdots at various pH from 4 to 11. The CVs (Figure 3) show a large catalytic current in acidic conditions that gets lower as the pH increases and then quickly drops off to zero between pH = 7 and pH = 8 (Figure S7). The current was confirmed to be from proton reduction by visible gas bubble formation at slower scan rates (Figure S8) and a hydrogen test from our previous study at pH = 4.^12^ The dropoff correlates well with the concentration of any acidic proton donor. At pH of 4 and 6, the current looks to be dominated by free protons, and the remaining current drops away at the p*K*_a_ value of dihydrogen phosphate at pH = 7.2 where it is a higher concentration than the free H^+^ ions. At higher pH values, the protons would come from H_2_O and the catalysis would have to swap from a water dissociation mechanism, which it seems unable to perform.

The experiments demonstrate that for reduced PFBT Pdots acting as a catalyst, there needs to be a stronger proton donor than water in the system. The catalytic behavior is also static in potential over the range of pH values that were tested and does not follow the reversible hydrogen electrode (Figure S9). This behavior indicates that the electrocatalysis relies on an initial reduction of the Pdots instead of a metal-based reaction, for example, a mechanism based on a Pd metallic catalyst. The role of the BT unit, therefore, needs to be considered because it will be easier to protonate upon reduction under acidic conditions compared to under basic conditions. If the protonation of BT is faster than the electron transfer to the residual Pd from reduced PFBT, then the protonation of BT is therefore a possible intermediate involved in the photocatalytic reaction. To compare how Pd-centered catalysis looks in the system of a Pd working electrode with the same area as the GC electrode ( 3 mm), the Pd-based mechanism radically differs from what is seen with the GC electrode (Figure S10).

**Figure 4:**
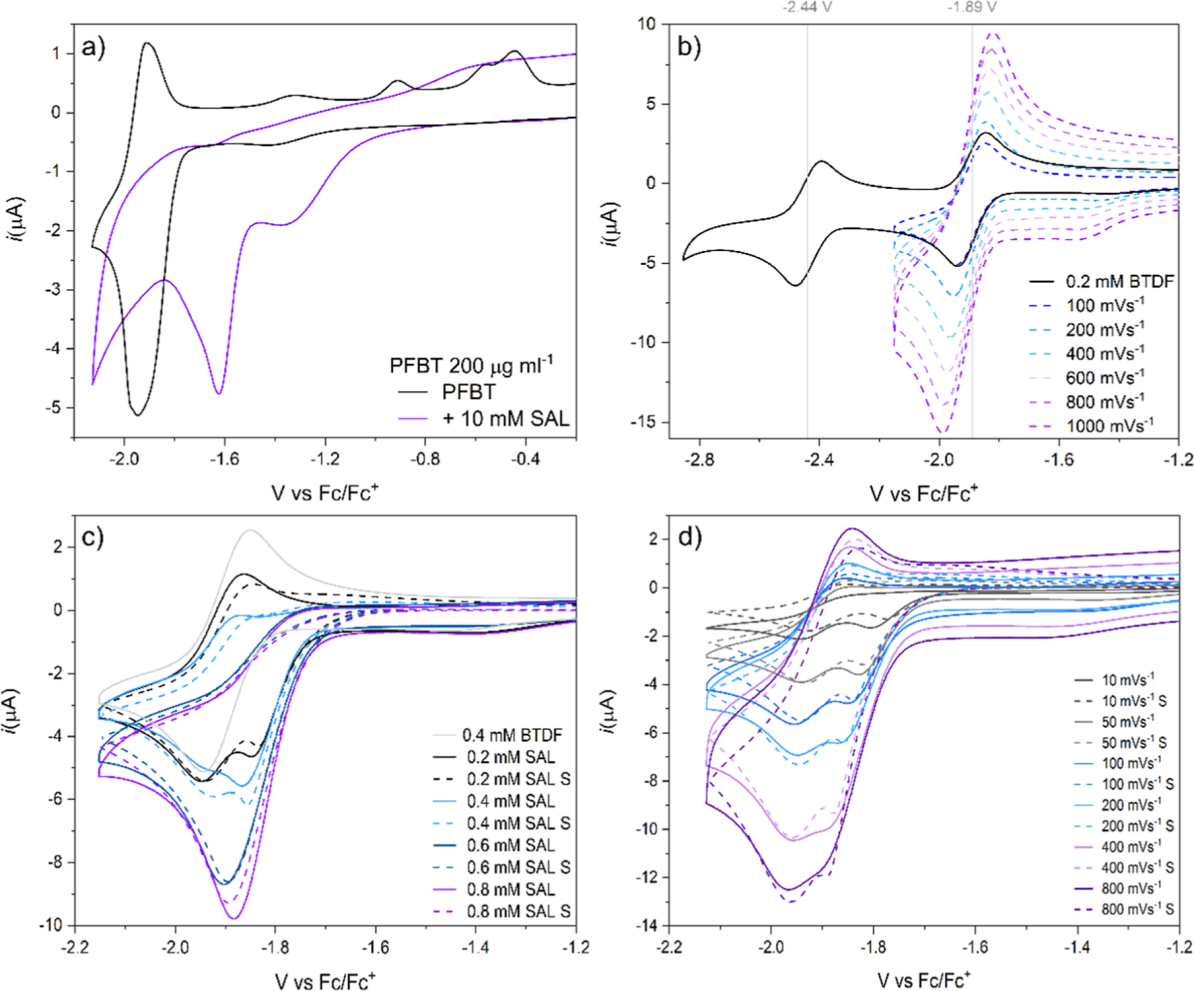
Cyclic voltammograms of BTDF and PFBT were recorded in THF. (a) CVs of the pristine PFBT polymer in THF at 200 μg mL^–1^ with and without the addition of a proton donor (salicylic acid) recorded at 100 mV s^–1^ (b) CVs of 0.2 mM BTDF with the *E*_1/2_ values and with varied scan rates with the current having a linear dependence on the square root of the scan rate. (c) Titration of 0.4 mM BTDF with salicylic acid (0.2–0.8 mM) and the corresponding simulated CVs (dashed lines). (d) CVs of 0.4 mM BTDF with 0.4 mM SAL with varying scan rates (10–800 mV s^–1^) and the corresponding simulated CVs (dashed lines).

In our previous study, we could monitor the protonation of the BT analog BTDN, from a CV in the presence of an organic acid, salicylic acid.^23^ We therefore first looked into the CV of pristine PFBT polymer both for the washed and the commercial as-received polymer while dissolved in THF. The electrochemical behavior of PFBT is very complex, with many available redox states (Figure S11). It is, however, clear that the reduced polymer reacts with acids in a solution (Figure 4a) by a positive shift of the wave, which is characteristic of a reaction following a reduction. To elucidate what kind of reaction is and calculate the rate of the protonation step, we designed and synthesized a model compound BTDF consisting of one BT unit and two fluorene units to mimic the PFBT polymer and use it for more in-depth electrochemistry studies.

### Electrochemical Properties of BTDF

BTDF shows a similar electronic structure to the PFBT polymer with the BT excitation and fluorene-related transition around 320 nm as well as a band from their interaction around 420 nm, both of these transitions are retained in the polymer but red-shifted due to the increased electron delocalization in the large conjugated system of the PFBT polymer^23,30−32^ (Figure S12). In comparison to the PFBT polymer, the redox behavior of BTDF is very clear with two reversible reductions at −1.89 and −2.49 V versus Fc/Fc^+^, and a linear scan rate dependence of the square root of the current as would be expected of a freely diffusing molecule. The two reversible reductions of BTDF are similar to that of the molecule BTDN (600 mV in between reductions as compared to 800 mV in BTDN), however, shifted to more negative potentials due to the difference in electron richness between the side groups. The electron-donating dioctylfluorene units make the BT unit more difficult to reduce as compared to the electron-withdrawing nitrile groups in BTDN.^23^

When adding salicylic acid as a proton donor to a BTDF solution in THF, there was a clear shift to the CV where the reduction becomes irreversible with a shift to more positive potentials and with an increase in current. In the conditions of the acids at the first reduction potential, BTDF did not perform catalysis for proton reduction (Figure S13). This can be explained by the high reductive potential that is probably required to reduce BTDF further which is the requirement for the proton reduction by BTDN on a glassy carbon electrode.^23^ However, with glassy carbon under this condition, there is not an electrochemical window to reduce BTDF further. The clean voltammetry behavior of BTDF at its first reduction potential is good enough for us to investigate the protonation mechanism of the BT unit of BTDF in detail.

The protonation mechanism of the BT unit can be extracted from the behavior of the shift in the first reduction wave. At high concentrations (more than two equiv) of acid, the wave is completely irreversible with a doubling in electric current. Including the positive shift, this is the mark of two consecutive follow-up reactions, a so-called EC (electrochemical step followed by a chemical step) reaction that is followed by a secondary reduction step either another electrode-based electron transfer (an ECE reaction: an EC reaction followed by a secondary electrochemical step) or an intermolecular charge transfer between the newly generated species similar to what happens in BTDN (a so-called EC-DISP reaction: an EC reaction followed by a disproportionate reaction).^33^ To disentangle the two types of reactions, acid titration and scan rate dependence experiments were performed (Figure 4c,d). During the titration, it can be seen that the reduction peak splits into two different peaks at lower acid concentrations and then merges at higher concentrations. At higher scan rates, a merge of the two peaks can be seen as well as a slight return of the oxidation peak showing that the irreversible nature of the reaction is dependent on the scan rate. The two data sets were then used to simulate the protonation mechanism.

**Figure 5:**
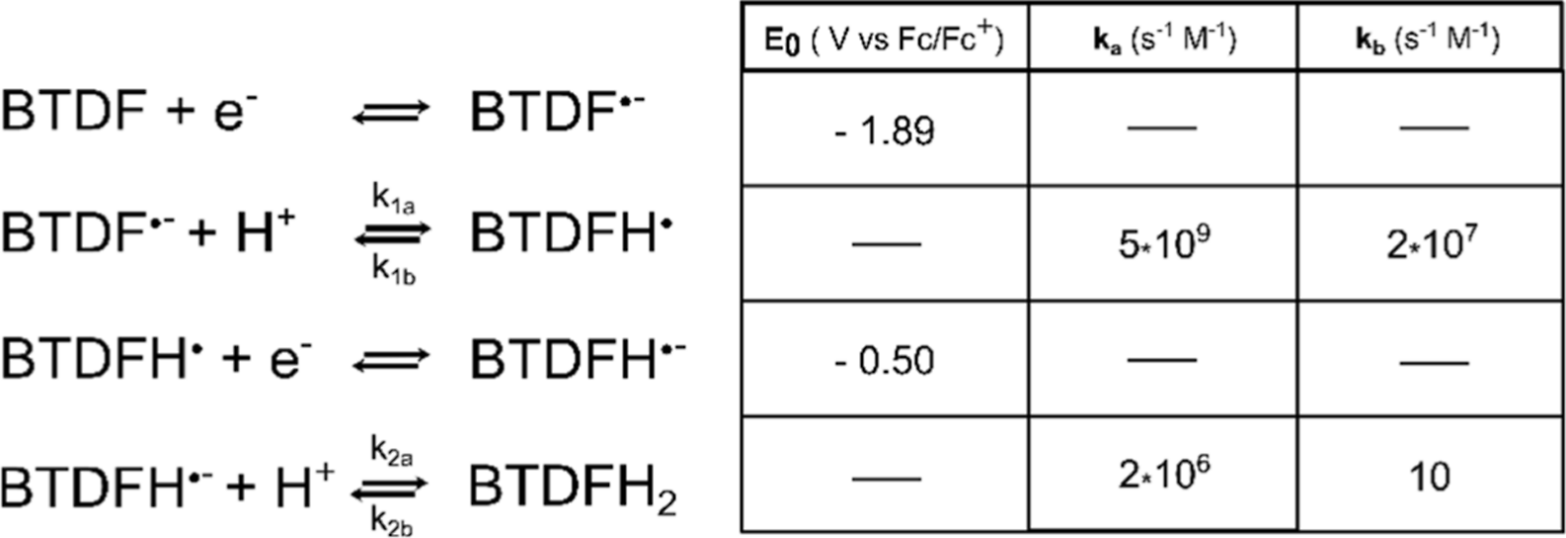
Simulated mechanism (left) and kinetic constant (right) of the reaction between reduced BTDF and protons.

Fitting a mechanism to the CV data (Figure 4c,d) shows that the overall BTDF reduction reaction follows an ECEC type reaction (an ECE reaction followed by a second chemical step) where the second reduction step occurs at a less reductive potential than the first reduction, −0.5 V compared to −1.89 V vs Fc/Fc^+^ in this case, but any value closer to zero than −1 V vs Fc/Fc^+^ fits the data quite well (Figure 5). A key feature to getting the peak-splitting and then merging at higher acid concentrations is that the first protonation is an equilibrium that is heavily shifted toward the protonated species with reaction kinetics around or exceeding the diffusion limit at 0.1–5 × 10^10^ M^–1^ s^–1^, but the back-reaction (2 × 10^7^ M^–1^ s^–1^) is still faster than the next protonation step 2 × 10^6^ M^–1^ s^–1^.

The result shows that the BT unit does react with protons at least once when it is reduced. As the chemical environments of BT in BTDF and PFBT are highly similar, it indicates that this protonation should happen in BT units in PFBT polymers as well when it is photochemically reduced.

### Exciton Quenching of Pdots by an Electron Acceptor

While the electrochemical studies show that the reduced PFBT polymer will react with sufficiently strong acids, the photochemical case does not have to be so straightforward. In the photochemical system, there is not a large pool of high-energy electrons to reduce the polymer; instead, the generated exciton will have to be reductively quenched by the SD to get the reduced polymer. However, the exciton can, in theory, dissociate in many different ways, not only reductively in PFBT, but both reductive quenching from DEA and oxidative quenching Pd particles have been previously reported.^27^ But the electron transfer from excited PFBT to Pd clusters via oxidative quenching has been reported as a main charge separation process for photocatalysis with PFBT in the presence of DEA.^27^

**Figure 6:**
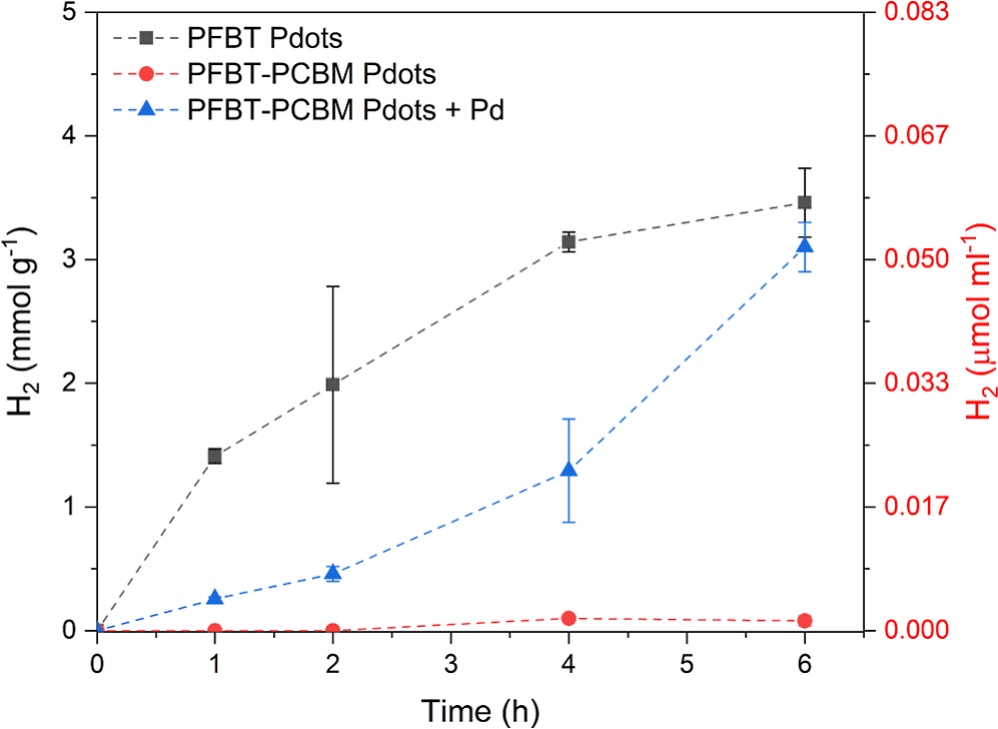
Photocatalytic hydrogen data for three different compositions of PFBT Pdots in pH 4 with ascorbic acid as the SD: unmodified PFBT Pdots (gray squares), PFBT–PCBM binary Pdots (red circles), and photodeposited Pd in PFBT–PCBM binary Pdots (blue triangles).

Oxidative quenching of PFBT by residual Pd has been reported up to at least 50% with 1170 ppm of Pd while still quenching around 25% with as low as 36 ppm Pd. However, the reductive quenching of PFBT by SDs to form reduced PFBT also seems to be an important pathway with 20–30% quenching in 30% DEA and 40% in ascorbic acid.^12,27^ We can also see a clear reductive quenching of PFBT in organic media with ascorbic acid (Figures S14–S15) since this is a condition with sufficiently acidic protons that the reduced polymer is expected to rapidly be protonated. This is even more likely with ascorbic acid as it will also release a proton as it decomposes after being oxidized and therefore works like a proton-coupled electron transfer (PCET) donor and not just a simple reductive agent.^34−36^ Therefore, it is also likely that electron transfer to an active Pd site could also happen from an already protonated reduced polymer.^37^ In this case, it is possible that the proton would transfer as well, as it has already been shown that BT sites will perform proton-coupled electron transfer reactions.^23^ This certainly requires that the residual Pd be close enough to the active site. It however generates the following question: what is the form of residual Pd in the PFBT polymer? It is hard to get direct spectral evidence from NMR or IR due to the low ppm level concentrations, except for the transient spectroscopic data.^27^ Instead, we used phenyl-C61-butyric acid methyl ester (PCBM) to extract electrons ultrafast from PFBT in binary PFBT:PCBM Pdots. If the residual Pd is a cluster acting as the catalytic site and the sole catalytic site, then the reduced PCBM generated after electron transfer from PFBT under light illumination should still be able to give electrons to the Pd cluster for catalysis as well, similarly to Pd clusters that can get electrons from the PFBT polymer. However, the photocatalysis was completely inhibited in the presence of PCBM, unless an extra Pd or Pt source is introduced afterward (Figures 6 and S16). This cannot be well explained by the unreachable Pd cluster by PCBM due to a well-embedded Pd cluster in PFBT. Moreover, we should still be able to observe hydrogen production anyway, because the Pd cluster could still get electrons after competing with PCBM from reduced or excited PFBT for catalysis due to fast electron transfer between PFBT to Pd (fs-ns).^27^ An explanation could be that the fast electron transfer from PFBT to PCBM inhibits the protonation of BT due to the short lifetime of reduced PFBT,^37^ therefore suppressing the proton reduction reaction.

Therefore, we hypothesize that the catalytically active residual Pd site should have a favorable interaction with the protonated BT unit. The protonated BT units in the reduced PFBT, at acidic conditions, work as both electron proton channels to the catalytic active sites. Another possibility is that the residual Pd is reduced by the reduced PFBT polymer and then protonated, as well. This species could then facilitate hydrogen–hydrogen bond formation with the protonated BT unit in another PFBT polymer for the following hydrogen formation. Notably, if involving the residual Pd is the sole catalytic pathway in PFBT Pdots, then the catalytically active Pd sites should be much less than the Pd amount detected by ICP, as is also evident from the comparable photocatalytic performance from the samples with 1000 and less than 10 ppm Pd. However, the pure organic catalytic process only based on protonated benzothiadiazole units cannot be ruled out based on this study. Notably, Pdots have a different chemical environment from the polymer dissolved in organic solvent, and the confined nanoparticle and large electric field formed between Pdots and water should also have a significant effect on the catalytic reaction, which still needs to be explored.

## Conclusions

In summary, we have studied the effect of chemical environments on the photocatalysis of PFBT Pdots and concluded that the protonation of benzothiadiazole (BT) in PFBT polymer could be an important intermediate process for photocatalysis under acidic conditions. From photocatalytic experiments, the PFBT sample with less than 10 ppm of Pd showed comparable photocatalytic activity to the sample with 1000 ppm. It indicates that the active residual Pd amount should be much less than the actual residual Pd in the polymer if Pd is the only catalytic site. With a model compound BTDF in electrochemical experiments, the reaction rate of protonation of the BT unit is found to be a fast and diffusion-controlled reaction. The results suggest that the protonated BT likely is an important intermediate of the PFBT polymer at acidic conditions, indicating a possible PCET process from the reduced protonated polymer to the intrinsic catalytic sites. This work therefore paves the road to understanding the reaction mechanism of polymeric photocatalysts with heteroatom block units. Further study on how the confined microenvironment in Pdots and the large electric field at the interface between Pdots and water affect the catalytic process will be helpful to further understand the photocatalytic process in Pdots.

## Supporting Information Available

Experimental procedures, characterization of products, spectroscopic and voltammetry data, and control experiments (PDF)
