# Supplementary material for: Role of the Benzothiadiazole Unit in Organic Polymers on Photocatalytic Hydrogen Production
Source: JACS Au. 2024 Jan 13;4(2):570–7. doi: 10.1021/jacsau.3c00681 (PMC10900483; doi:10.1021/jacsau.3c00681)
Supplement: Supplementary file 1 — au3c00681_si_001.pdf [file au3c00681_si_001.pdf]

# Supporting Information

## The Role of the Benzothiadiazole Unit in Organic Polymers on Photocatalytic Hydrogen Production

Martin Axelsson,† Ziyang Xia,‡ Sicong Wang,† Ming Cheng,*,‡ and Haining Tian*,†

† Department of Chemistry-Ångström Laboratory, Uppsala University, Uppsala 75120, Sweden

‡ Institute for Energy Research, Jiangsu University, Zhenjiang 212013, China

E-mail: mingcheng@ujs.edu.cn; haining.tian@kemi.uu.se

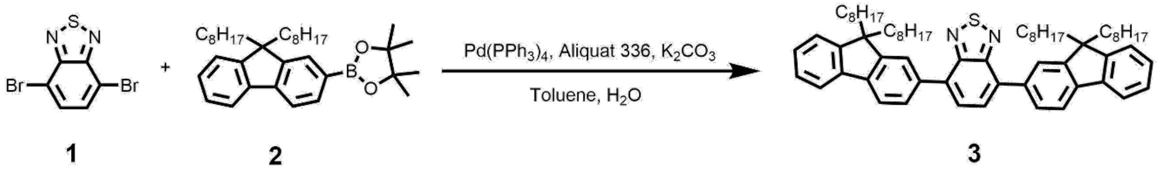


Scheme S1: The scheme for the synthetic procedure of BTDF

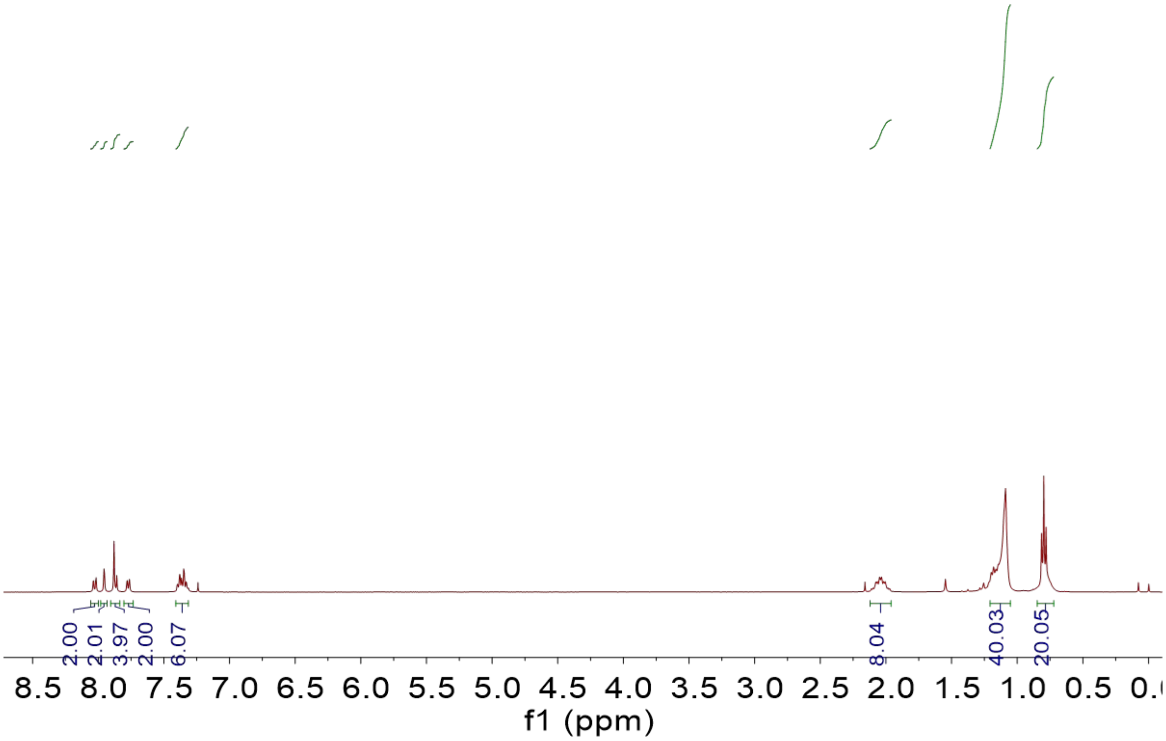


Figure S1: 1H NMR (CDCl3) spectrum of BTDF

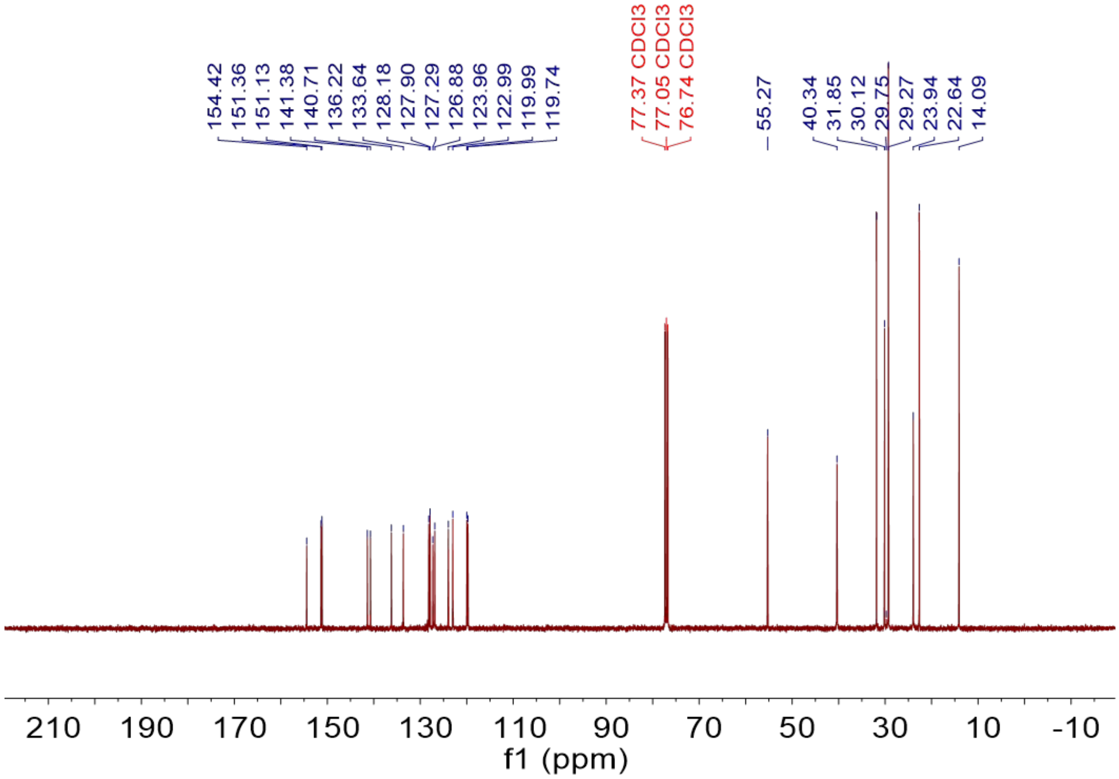


Figure S2: 13C NMR (CDCl3) spectrum of BTDF

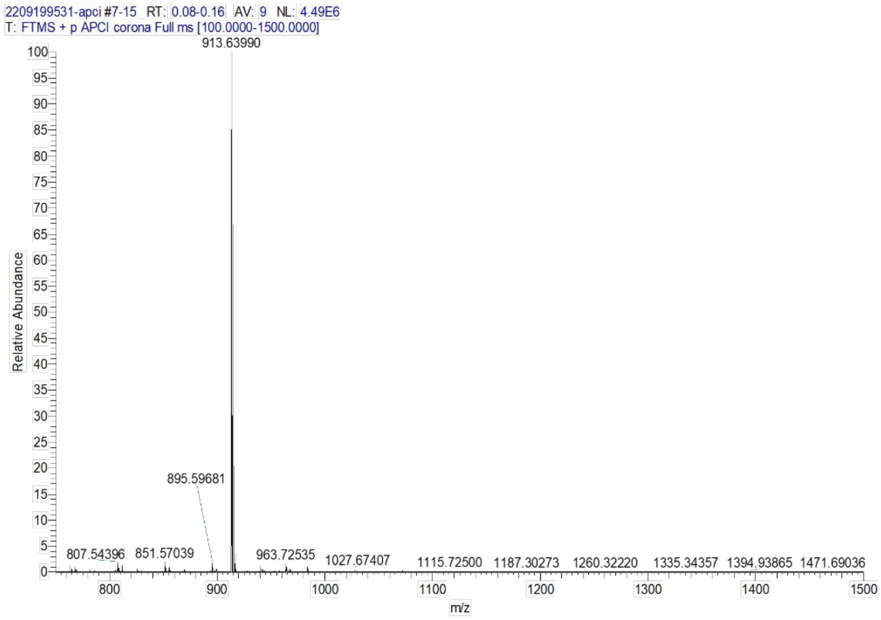


Figure S3: HRMS-EIS of BTDF high retention, the peak of 913.6 m/z fitting the calculated mass of BTDF

**Photocatalysis**

The Pdots were controlled with UV-Vis (Figure S4) and measured to a difference of 3% OD. The particle sizes were measured using DLS and the <10 ppm Pd particles had an average hydrodynamic radius of 55.67 nm while the 1000 ppm Pd particles had an average hydrodynamic radius of 49.36 nm a difference of 11.4%. The quantum yields of samples with 1000 ppm Pd and 10 ppm Pd in presence of ascorbic acid were determined to be 0.44% and 0.31% at 445 nm, respectively, based on the quantum yield test in our previous work[1].

The photocatalytic $H_2$ experiment with DEA at an adjusted pH of 10.9 to be closer to the pKa value of DEA at pH 10.98 (Figure S5). No significant difference was seen between the two pH values, indicating that the main difference between the two SD conditions is not because the Ascorbic acid is close to its pKa while the DEA is not.

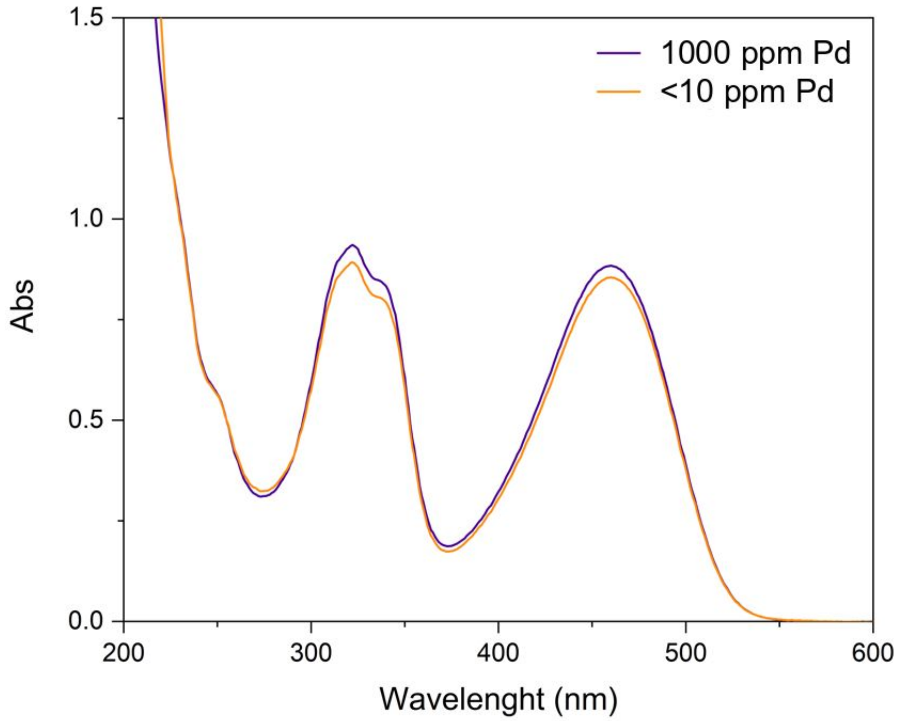


Figure S4: UV-Vis comparison of the two Pdot batches with different Pd concentrations

with a 3% difference in absorption at 457 nm.

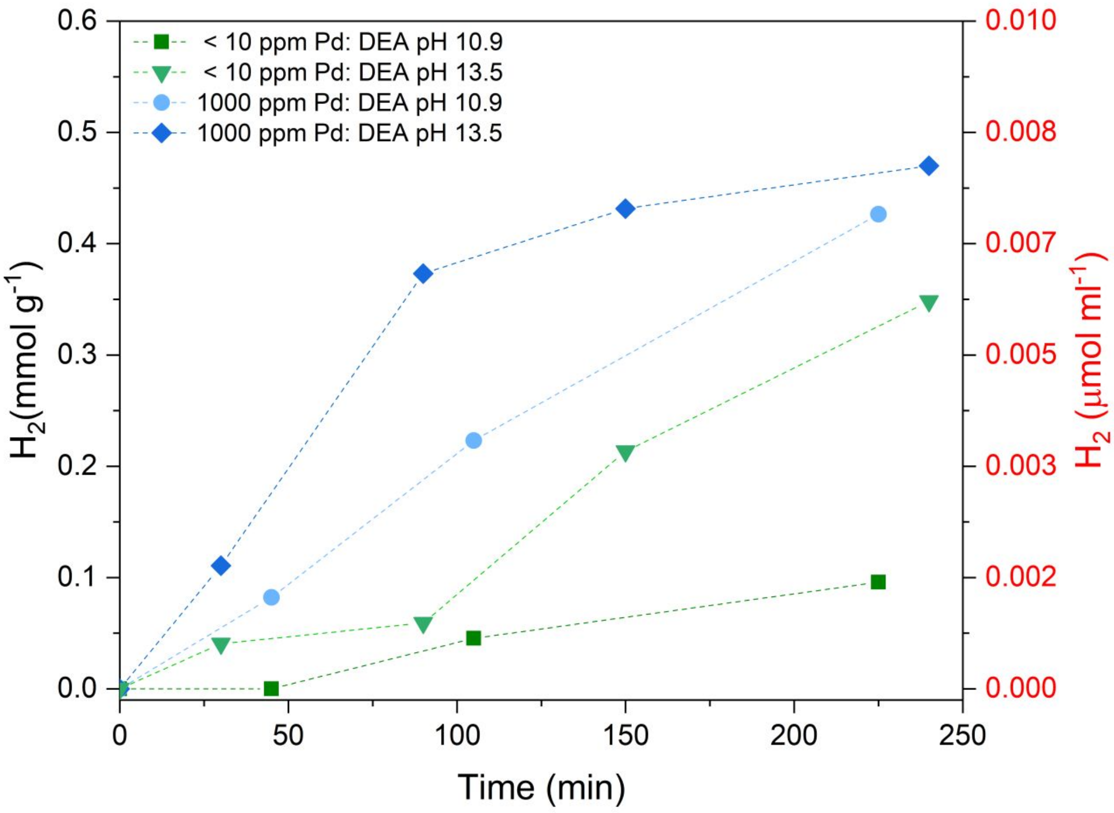


Figure S5: The photocatalytic GC data for the four conditions of hydrogen evolution from PFBT Pdots with DEA, both at uncorrected pH values and close to the pKa of DEA.

6

**Electrocatalysis and pH dependence**

CVs of the buffer backgrounds over the measured pH span (Figure S6 (left)) and the comparison between the backgrounds and the Pdots in the acidic conditions (Figure S6 (right)). A closer look at the pH values between pH 7 and pH 8 where the catalytic current drops off at a slower scan rate of 10 mVs$^{-1}$ for higher detail of the curve (Figure S7). Figure S9 shows that the catalytic current does not follow an onset dependent on RHE, instead the static onset potential seen in Figure 2 is attributed to an initial reduction of the polymer that is static vs NHE. Figure S8 Showing bubble formation while scanning at 10 mVs$^{-1}$ both as a disturbance in the CV and from a picture of the experiment, the bubble is assigned to be $H_2$ formation as reported in literature 1.

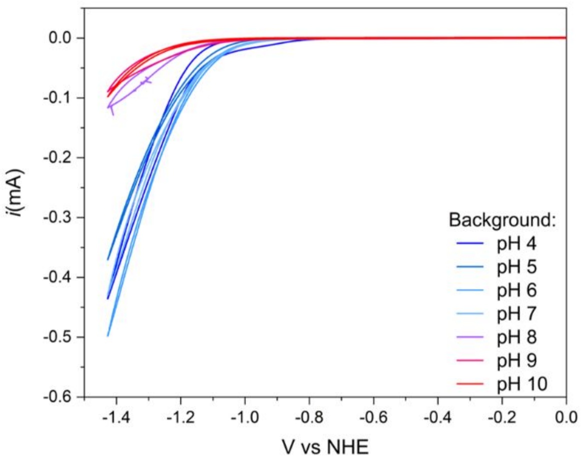

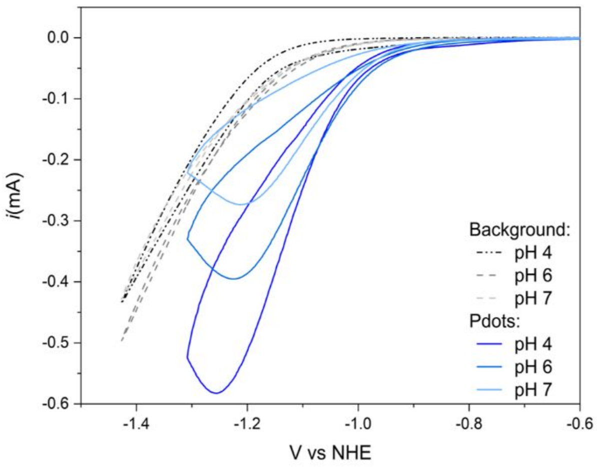


Figure S6: Backgroud CVs for the electrocatalytic experiments with PFBT Pdots of the experiment recorded at 50 mVs$^{-1}$ in 50 mM phosphate buffer and 50 mM KCl as the supporting electrolyte, Over the full pH range (left) and from pH 4 to pH 7 with the Pdot CVs as a comparison(right)

Comparing the two electrodes (Figure S10), the Pd in the basic condition has an onset potential at about -0.6 V vs NHE which we attribute to the proton reduction with water as a proton source. This reduction wave still appears before that of the proton reduction from PFBT at the GC electrode but no trace of a catalytic signal can be detected in the CV from this system. At the acidic condition there is also a pre-wave with an onset at about -0.3 V vs NHE this is attributed to the direct reduction of protons or alternatively the reduction of $H_2PO_4^-$ which are both limited sources

of protons in the system.

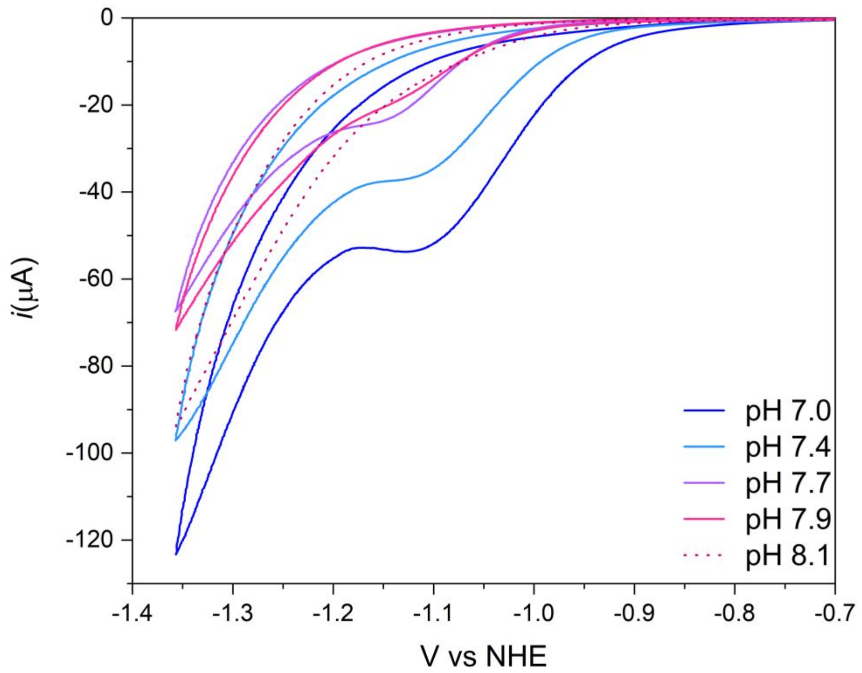


Figure S7: CVs of the pH dependence of electrocatalytic hydrogen evolution from PFBT Pdots, in the range of pH=7 to pH=8 recorded at 10 mVs⁻1 in 50 mM phosphate buffer and 50 mM KCl as the supporting electrolyte.

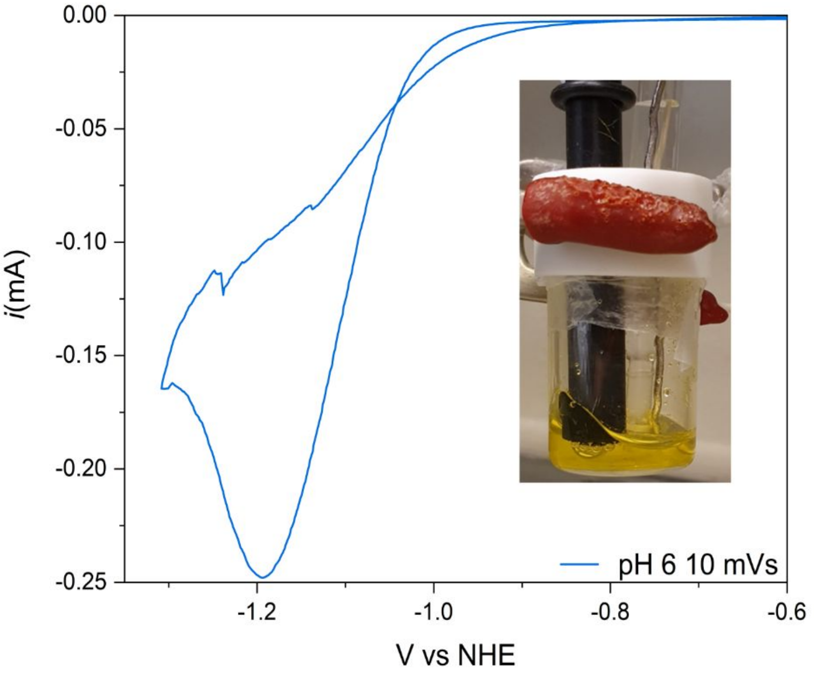


Figure S8: CVs of the pH dependence of electrocatalytic hydrogen evolution from PFBT Pdots, at pH 6 recorded at 10 mV s$^{-1}$ in 50 mM phosphate buffer and 50 mM KCl as the supporting electrolyte, with a photo of the electrochemical cell with visual bubble formation.

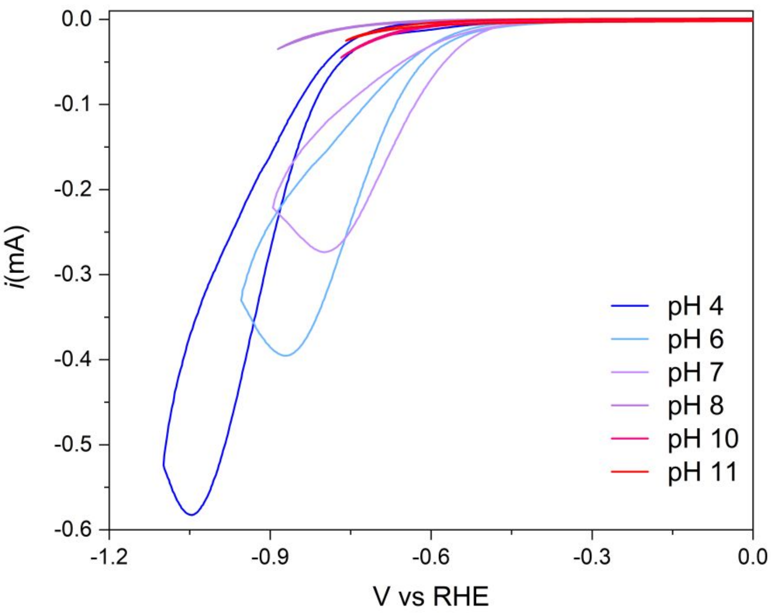


Figure S9: CVs demonstrating the pH dependence of electrocatalytic hydrogen evolution from PFBT Pdots vs RHE, in 50 mM phosphate buffer and 50 mM KCl as the supporting electrolyte. Showing that the onset of catalysis does not follow RHE but instead is static vs NHE in this range.

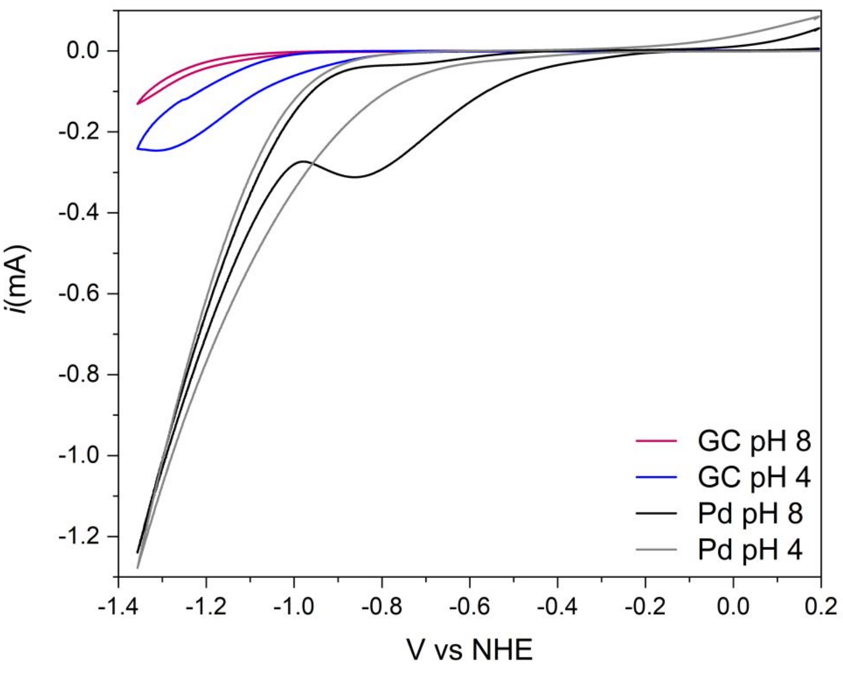


Figure S10: CVs demonstrating the pH dependence of electrocatalytic hydrogen evolution from PFBT Pdots, in 50 mM phosphate buffer and 50 mM KCl as the supporting electrolyte. The CVs of the Pdot solutions in pH 4 and 8 with a GC 3 mm disc electrode and a Pd 3 mm disc electrode at 100 $mVs^{-1}$.

Electrochemical properties of BTDF

Figure S11 shows the complex redox behaviour of PFBT especially on the second scan of the CV when the polymer has been reduced at least once. Figure S12 shows the UV-Vis absorption spectra of PFBT and BTDF indicating that their electronic structures are quite similar except for the red-shifted absorption in the polymer, likely due to an increased conjugated system[2–4]. Figure S13 shows that the reduction of SAL on the glassy carbon surface occurs before the second reduction of BTDF, hence no BTDF-based catalysis can be seen in this system.

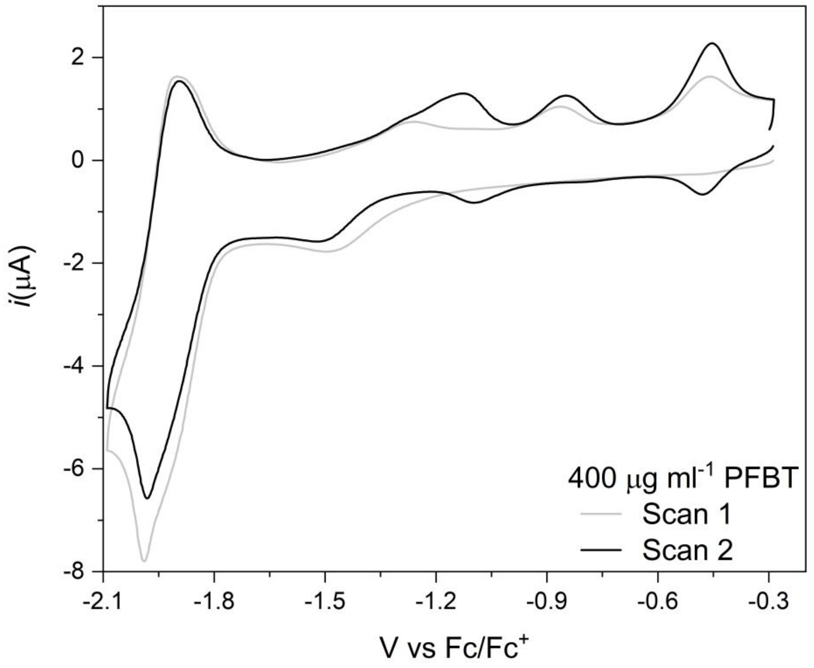


Figure S11: Showing the CV of PFBT in THF, 1st and 2nd scan at 100 $mVs^{-1}$. Demonstrating the many redox states available in PFBT after it has been reduced once.

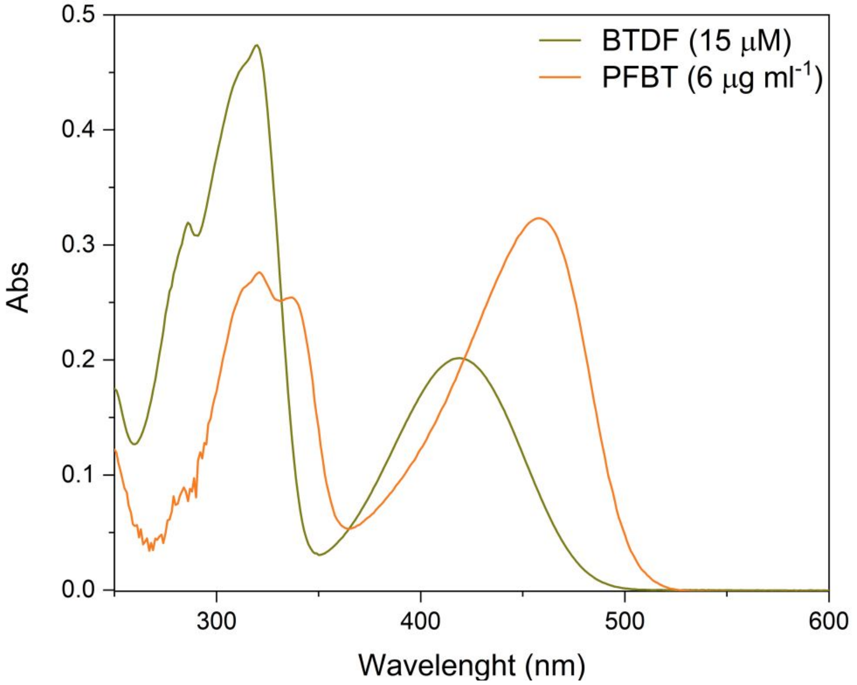


Figure S12: Showing the UV-Vis absorption spectra of BTDF (15μM) and PFBT (6 μg ml−1) demonstrating the similar transitions and the red-shift that occurs in the polymer.

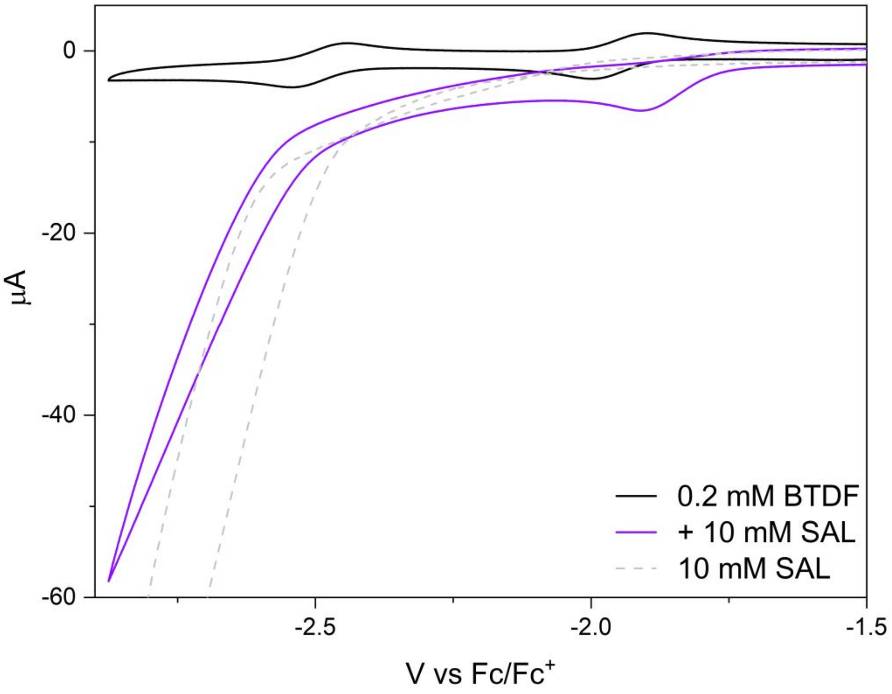


Figure S13: Showing the CVs of salicylic acid and BTDF with salicylic acid in a wide scan. Shows that the reduction of SAL on the glassy carbon occurs before the second reduction of BTDF.

## Exciton Quenching of Pdots by an Electron Donor

Figure S14 shows the fluorescence quenching data showing that reductive quenching is possible between the pristine PFBT polymer and Ascorbic Acid also in organic solvents. The slow quenching seen in the Stern Volmer plot (Figure S15) is attributed to a likley hindered decomposition pathway for oxidised Ascorbic Acid in THF as compared to water as well as limited solubility of the acid in THF[5,6].

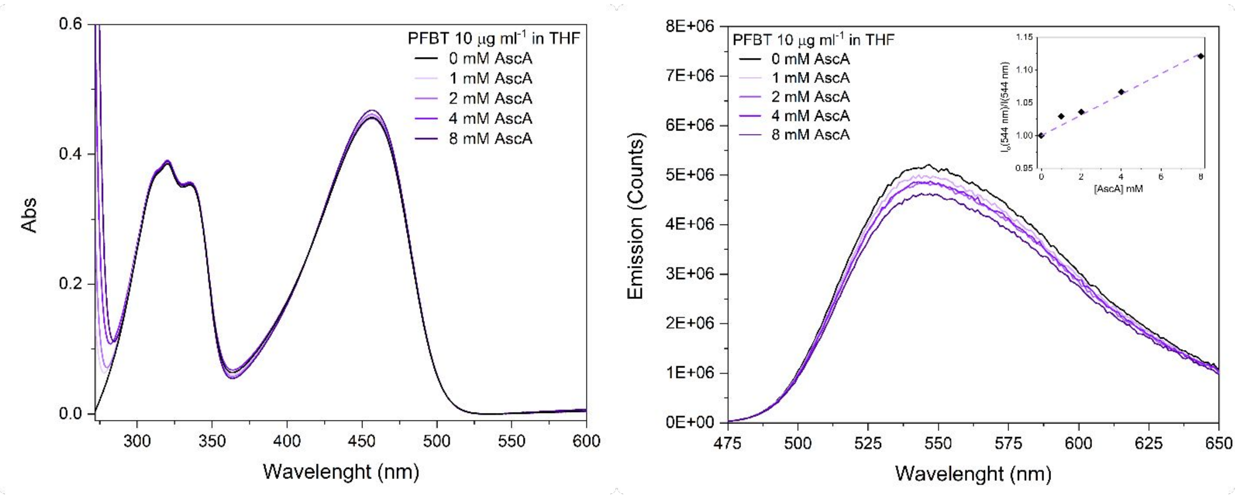


Figure S14: The steady-state fluorescence quenching of PFBT with Ascorbic Acid in THF. Showing the absorption spectra of PFBT with the addition of Ascorbic Acid (left) and the corresponding emission spectra (right) and the Stern-Volmer plot showing the quenching as a linear slope in the range probed (inset).

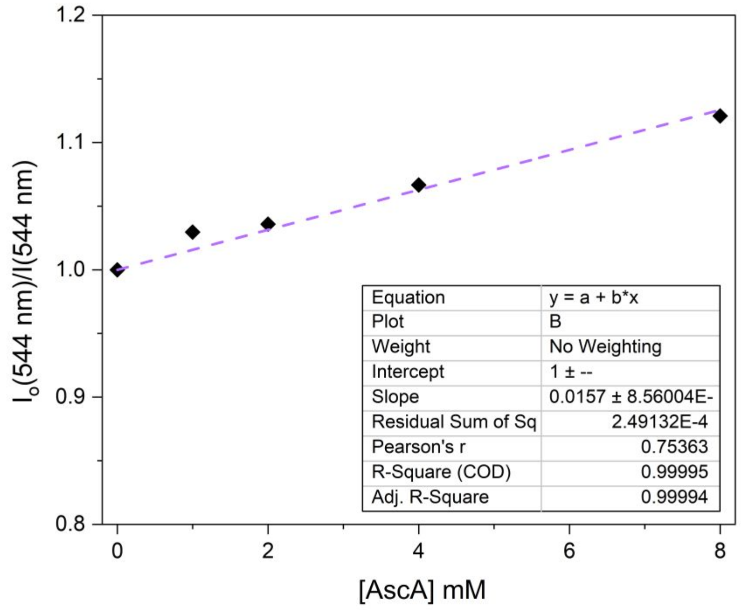


Figure S15: The Stern-Volmer plot for fluorescence quenching of PFBT with Ascorbic Acid, showing the quenching as a linear slope and the linear fit.

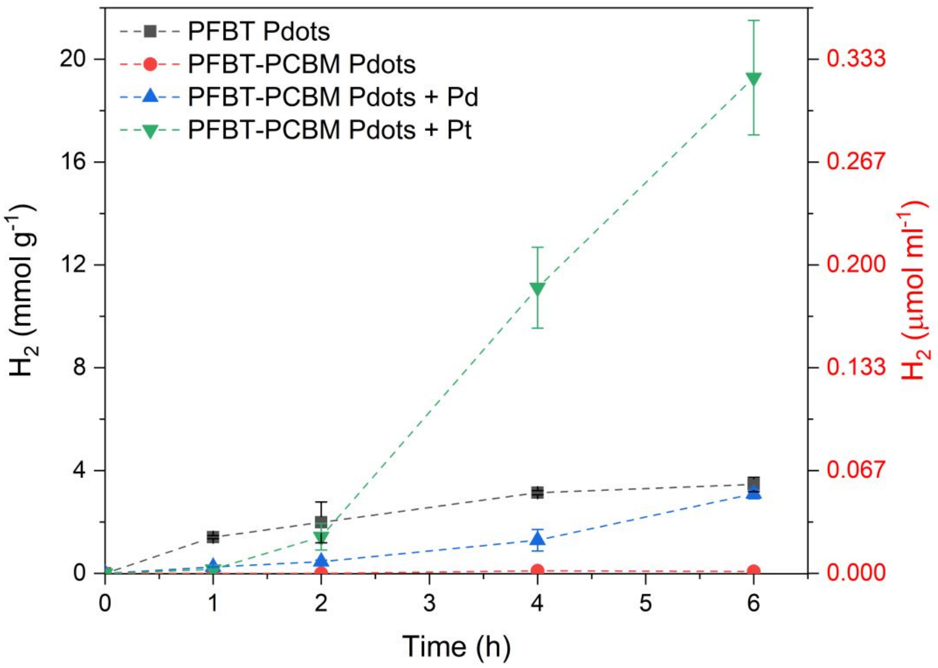


Figure S16: The photocatalytic GC data for four different compositions of PFBT Pdots in pH 4 with ascorbic acid as the SD. Showing that unmodified PFBT Pdots (grey squares), binary Pdots with added PCBM as an electron acceptor (red circles), Pdots with added PCBM as well as photo deposited Pd (blue triangles), and Pdots with added PCBM as well as photo deposited Pt (green triangles) for hydrogen evolution.

## References

(1) Wang, L.; Fernández-Terán, R.; Zhang, L.; Fernandes, D. L. A.; Tian, L.; Chen, H.; Tian, H. Organic Polymer Dots as Photocatalysts for Visible Light-Driven Hydrogen Generation. Angew. Chem. Int. Ed. 2016, 55, 12306–12310.

(2) Griffiths, J. Colour and constitution of organic molecules; Academic Press, 1976.

(3) Rogers, J. E.; Nguyen, K. A.; Hufnagle, D. C.; McLean, D. G.; Su, W.; Gossett, K. M.; Burke, A. R.; Vinogradov, S. A.; Pachter, R.; Fleitz, P. A. Observation and Interpretation of Annulated Porphyrins Studies on the Photophysical Properties of meso-Tetraphenylmetalloporphyrins. J. Phys. Chem. 2003, 107, 11331–11339.

(4) Hanson, K.; Roskop, L.; Djurovich, P. I.; Zahariev, F.; Gordon, M. S.; Thompson, M. E. A Paradigm for Blue- or Red-Shifted Absorption of Small Molecules Depending on the Site of π-Extension. J. Am. Chem. Soc. 2010, 132, 16247–16255.

(5) Pellegrin, Y.; Odobel, F. Les donneurs d’électron sacrificiels pour la production de combustible solaire. C. R. Chim. 2017, 20, 283–295.

(6) Lakowicz, J. R. Principles of Fluorescence Spectroscopy Joseph R . Lakowicz, 3rd ed.; Springer Science and Business Media, 2006.

18
